# Mr. Brudenell Carter, F.R.C.S.

**Published:** 1918-11-02

**Authors:** 


### MR. BRUDENELL CARTER, F.R.C.S.

### A Man of Principle in Medical Practice.

A doctor once told the present writer that the husband's point of view at the confinement of a wife was always regard for her welfare, and by comparison indifference to that of the child. This observation is supported by the behaviour of the late Mr. Brudenell Carter's father, whose wife died on the child's birth, October 2, 1828. Mr. Carter, senior, abandoned all interest in his son, who was not expected to live, and the child owed its christening, and, indeed, its names, to the sixth Earl of Cardigan, a family friend, who had the boy baptised "Robert Brudenell." Educated at the London Hospital, after, it seems, an apprenticeship to a fully-qualified practitioner, an experience which he rated highly, he qualified in 1852. Only a year later he showed his literary and medical gifts by an astonishing performance—namely, the publication of his "Pathology and Treatment of Hysteria." The balance of mind, the grasp of principle, the alertness of observation, and the vivid style show once more that wisdom is the gift, not of age, but of capacity for experience. Brudenell Carter made his mark, but not his fortune, and turned to private practice for a livelihood. Not too eager for this particular work, he seized the chance offered by the Crimean War, and became a staff surgeon in Turkey. During his work there he met the then correspondent of the *Times*, and wrote, on the latter's suggestion, a series of letters to that journal, whose publication once more showed his talent and aroused attention. Various moves after his return to England when the war was over led him to settle at Nottingham, and, though engaged not too happily in general practice, he began his study of diseases of the eye, and coupled with it much literary work on nervous conditions and the relation of educational systems to health. The mere titles of his volumes on these subjects are remarkable. "The Artificial Production of Stupidity," "The Influence of Education in Preventing Nervous Diseases," "The Physiological Influences of Certain Methods of Teaching." The mind which could evolve important works upon these subjects was obviously better fitted for scientific pursuits than to the practice of the art of medicine, which is the pre-occupation, and rightly so, of general practice. Hence it is not surprising that Mr. Brudenell Carter did not succeed in settling in general practice, or that, after a removal to Stroud, where he entered into a partnership, he should gravitate to London and devote himself to ophthalmic surgery. He took the precaution, on making this plunge, of reopening his relation with the *Times*, and he became a member of the staff. At St. George's Hospital he found his feet, and, the work being congenial, he was able to devote his spare hours to further medical composition. He gave a new life to the dry material of text-books, and his technical works were distinguished as much for the quality of their writing as for their practical use to students. These works were crowned in 1887 by the publication of his "Manual of Ophthalmic Surgery," and by this time honours began to crowd upon him. Mr. Carter was a valued and very constant member of our staff from 1897 to 1906. His was the pen of a ready writer, and he was always prepared to co-operate heartily to the fullest extent of his powers, which were great, and most helpful and valuable. After twenty-eight years on the staff of St. George's Hospital he retired in 1898, and was promoted—oddly enough, because of the ancestral claims accorded to the heraldic researches undertaken by him from motives of personal interest in his family history—to the rank of a Knight of Justice in the Order of St. John of Jerusalem. His last Encyclical, for so it deserves to be called, was "Doctors and their Work," published in 1903. This book made a success, and was remarkable for its insistence on the value of apprenticeship, which, as a system of training in medicine, has gone out of fashion with the various official means of qualification now codified in several Acts of Parliament. A nonagenarian, like Florence Nightingale, he resembled her in his keen eye for principles and his belief in reality rather than the latest arrangement in medical education. As a medical thinker he has his own place in the Pantheon of modern medicine.

